# Use of fertility awareness methods as a component of safer conception for women in HIV-serodifferent relationships in Kenya

**DOI:** 10.1186/s12978-021-01128-5

**Published:** 2021-04-01

**Authors:** Yasaman Zia, Jennifer Velloza, Lynda Oluoch, Richard Momanyi, Sarah Mbugua, John Njoroge, Stephen Gakuo, Edwin Mugo, Nicholas Thuo, Catherine Kiptinness, Njambi Njuguna, Kenneth Ngure, Nelly R. Mugo, Renee Heffron

**Affiliations:** 1 Department of Global Health, University of Washington, 325 Ninth Avenue, Box 359927, Seattle, WA 98104 USA; 2 Department of Epidemiology, University of Washington, Seattle, WA USA; 3 Kenya Medical Research Institute, Nairobi, Kenya; 4 Department of Community Health, Jomo Kenyatta University of Agriculture and Technology, Nairobi, Kenya; 5 KAVI Institute of Clinical Research, University of Nairobi, Nairobi, Kenya

**Keywords:** HIV, Family planning, Conception, Fertility

## Abstract

**Background:**

For couples affected by HIV, and serodifferent couples in particular, pregnancy desire is often juxtaposed against the risk of HIV transmission between the couple and the potential neonate leading to thinking about measures to minimize risk of HIV transmission. We assess the use of fertility awareness methods [FAM] and evaluate the drivers of alignment between indicators of fertility and sexual behavior among HIV-serodifferent couples desiring pregnancy.

**Methods:**

HIV-serodifferent couples from Thika, Kenya were enrolled into an open-label pilot evaluation of safer conception strategies. Women responded to daily 7-item short message service [SMS] surveys on FAM and sexual activity. Menstrual cycles were categorized as having condomless sex aligned, not aligned, or partially aligned to the predicted peak fertility. We used binomial logit models with generalized estimating equations to assess alignment between condomless sex during peak fertility days and FAM results. We used Cox proportional hazards to compare pregnancy incidence among months with sex and peak fertility aligned and mis-aligned.

**Results:**

A total of 6929 SMS surveys across 252 menstrual cycles of 65 women were included. Reporting “sticky” cervical mucus (adjusted odds ratio [aOR]: 2.25, 95% confidence interval [95% CI]: 1.30, 3.90) and positive ovulation prediction kit [OPK] result (aOR: 2.07, 95% CI: 1.11, 3.86) were associated with increased likelihood of alignment of condomless sex during peak fertility. Pregnancy incidence was statistically similar among periods with sex aligned and not aligned with peak fertility.

**Conclusions:**

Among women engaged in a comprehensive safer conception program, a moderate percentage of women aligned condomless sex and predicted peak fertility days at least once. While FAM, particularly cervical mucus and OPK, are an inexpensive option for couples to consider using as a component of their safer conception strategies, antiretroviral-based strategies remain important to minimize risk.

## Plain English summary

HIV-serodifferent couples are partnerships where one partner is living with HIV and the other is not. For these couples, the desire to become pregnant can be in contrast to the risk of transmitting HIV either to the baby or between the couple. Safer conception strategies can be used to reduce the risk of HIV transmission during pregnancy attempts, and they include fertility awareness methods [FAM] which involve timing condomless sex to the time when a woman is most likely to become pregnant during her menstrual cycle, called the peak fertility window. FAM include tracking menstrual cycles, monitoring cervical mucus, measuring daily body temperature, and using an ovulation prediction kit [OPK].

Seventy-four HIV-serodifferent couples were enrolled in a clinical study in Kenya. Each wanted to become pregnant and all were trained to track FAM. Women reported their sexual activity and FAM indicators via text message every day.

Sexual activity was better aligned to the peak fertility window when women found their cervical mucus to be “sticky” and if they had a positive OPK result. The use of these FAM indicators to align condomless sex with peak fertility did not affect pregnancy outcomes.

While FAM indicators are an inexpensive option for HIV-serodifferent couples to consider using as a part of their safer conception strategies, other HIV prevention strategies remain important to minimize risk.

## Background

For heterosexual couples who desire pregnancy, augmenting the probability of conception by estimating days with peak fertility and having sex on those days is an important method to achieve reproductive goals. Fertility awareness methods (FAM) are used to identify peak fertility days and optimally time condomless sex to these days to improve chances of conceiving [1]. For people wanting to avoid pregnancy, FAM is also used to indicate days to avoid condomless sex [2–4]. However, predicting peak fertility and ovulation can be challenging [3, 4] for couples and medical providers alike [5]. Low-cost objective FAM methods are self-administered and tracked by women to estimate predicted peak fertility days: cervical mucus quality, first presence of luteinizing hormone in urine, start of menses, and changes in basal body temperature. Additionally, low technology mobile applications for tracking FAM and sexual activity have been observed to improve patient–provider communication and women’s knowledge around fertility [6, 7].

For couples affected by HIV, and serodifferent couples in particular, pregnancy desire is often juxtaposed against the risk of HIV transmission between the couple and the potential neonate leading to thinking about measures to minimize risk of HIV transmission. HIV-serodifferent couples can employ any number of “safer conception” strategies to reduce HIV risk during pregnancy attempts, including timing of condomless sex to peak fertility and use of condoms at all other times, antiretroviral therapy (ART), pre-exposure prophylaxis (PrEP), and/or reproductive assistance technologies when fertility may be compromised [8–10]. Tailoring these interventions as couples’ needs change over time requires adaptability on the part of the program or provider and frequent discussion about fertility intentions [8, 11].

Several studies have assessed the use of FAM indicators to improve the time-to-pregnancy [12, 13], and have often been used for couples presenting with fertility challenges. But few of these studies have occurred in Africa where fertility rates and pregnancy desires are some of the highest globally and up to 50% of people living with HIV have been estimated to have an HIV-negative partner [14, 15]. The FAM indicators present an optional strategy for safer conception to optimally time condomless sex. Among African HIV-serodifferent couples engaged in a comprehensive safer conception program, we assessed daily tracking of FAM and how fertility indicators aligned with condomless sex, predicted peak fertility windows, and ultimately pregnancy incidence.

## Methods

The Safer Conception Intervention for Partners (SCIP, clinicaltrials.gov #NCT03030768) was an open-label pilot evaluation of a comprehensive safer conception package that enrolled 74 heterosexual HIV-serodifferent couples between March 2016 to April 2018 in Thika, Kenya [16]. Eligible couples were sexually active (six or more sex acts together in previous three months), had immediate fertility intentions, had no indication of clinically determined subfertility or infertility, and had access to a personal mobile phone. Couples were followed monthly until pregnancy and quarterly during pregnancy.

A comprehensive package of safer conception methods was offered to each couple and the couple selected methods that met their preferences, including: antiretroviral therapy (ART) for the partner living with HIV; daily pre-exposure prophylaxis (PrEP, co-formulated emtricitabine/tenofovir disoproxil fumarate (FTC/TDF)) for the HIV-negative partner; instruction and counseling about timing sexual activity to peak fertility periods; referrals for voluntary medical male circumcision for HIV-uninfected men; referrals for sperm washing, self-insemination, and other assisted reproductive services; and testing and treatment for sexually transmitted infections (STIs) [6, 16]. In SCIP, there were no HIV transmission events and the 12-month cumulative pregnancy rate was 61.8% among all 74 couples with approximately half becoming pregnant [16].

### Fertility awareness methods

All couples were counseled on the benefits for reducing HIV risk if condomless sex was timed to predicted peak fertility periods and condoms were used outside of peak fertility periods. Condoms were provided to all couples during all visits. To increase the awareness of peak fertility, education about FAM indicators and how to track them was provided to women throughout the study. FAM counseling included demonstration and practice with at-home ovulation prediction kits (OPK; Clearblue by Swiss Precision Diagnostics GmbH), digital thermometers to measure basal body temperature (BBT) daily, and assessment of cervical mucus, and menstruation. OPK and thermometers were provided to women by the study. For OPK, women were instructed to use the kit daily for a 10-day period starting 10 days after each menstrual cycle began. For cervical mucus assessment, women were taught about four different qualities of cervical mucus and that the one indicating peak fertility was when mucus is “sticky like egg whites.”

### Data collection

Data on demographics, partnership characteristics, and fertility intentions were collected in clinic by research staff using paper-based case report forms at baseline and follow-up study visits. These data were transcribed into REDCap, a web-based data management software. Daily SMS surveys were used to capture data on four fertility awareness methods: BBT, whether cervical mucus was sticky, results from OPK, and whether there was menstrual bleeding that day. The SMS survey also captured daily data on sexual activity and condom use. Daily SMS messages were sent and managed by mSurvey, a Nairobi-based mobile data collection company (https://msurvey.co/).

### Menstrual cycle and peak fertility calculations

Using a women’s menstrual history (including retrospectively captured data at enrollment if it was known and data collected during follow up), study staff predicted each woman’s upcoming peak fertility days and both partners received a weekly SMS reminder with the dates of her upcoming peak fertility. Peak fertility was calculated as the 4-day interval occurring immediately prior to the next predicted ovulation day. The ovulation day was estimated using the woman’s average cycle length and OPK results. The algorithm to predict peak fertility periods required data from either two to three menstrual cycles and 1 to 2 months of OPK results or three or more menstrual cycles. Thus, women did not have peak fertility predicted until sufficient data were captured. Peak fertility predictions were not provided if there was only one menstrual cycle reported and no OPK result, or if anovulation was indicated through negative OPK results every day during three consecutive menstrual cycles.

Menstrual cycle dates were calculated for each predicted peak fertility period using the woman’s average self-reported menstrual cycle length and imputing follicular and luteal phases from the predicted peak fertility days. If visits were skipped and data on menstrual cycle length were not available, the average menstrual cycle length for women by age group was used to calculate menstrual cycle dates [17] (2% of cycles). If predicted peak fertility periods overlapped, (indicating mismeasurement of fertility days), only the second imputed menstrual cycle was included for analysis (22% of cycles).

### Alignment outcome

Menstrual cycle months were categorized as having condomless sex aligned to peak (sex only during peak fertility days), not aligned to peak (sex only outside of peak days), or partially aligned to peak (sex within and outside of peak days) fertility. In a sensitivity analysis, we widened the peak fertility period by 4 days (2 days prior and 2 days after the primary window).

### Statistical methods

SMS data were included if they were reported during menstrual cycle months that had a corresponding predicted peak fertility period and occurred prior to the menstrual cycle when a woman’s first pregnancy occurred. SMS data were included for inferential analysis if they were collected subsequent to a woman’s second month in the study (to allow sufficient time for women to accurately report their FAM indicators). Descriptive statistics and graphics were used to summarize women’s menstrual cycle alignment overall and over time. To assess which fertility indicators (mucus, menses, OPK) were associated with alignment of sexual activity and peak fertility after baseline visits, we used binomial logit models using generalized estimating equations for repeated measures to assess whether condomless sex was associated with having an indication of high fertility. While we collected data on BBT, we excluded it from this analysis because it indicates that ovulation has just passed, rather than being an indicator of immediate ovulation [18, 19].

Across menstrual cycle months, multinomial logit models using generalized estimating equations for repeated measures evaluated characteristics of women and couples whose sexual activity aligned to predicted peak fertility period compared to those who partially or completely mis-aligned sex and peak fertility. Since visits to the research clinic were not parallel with the beginning of a new menstrual cycle, we conducted a sensitivity analyses using data lagged forwards and backwards by one visit when it pertained to personal and partnership characteristics that may change over time. Pregnancy incidence among those whose sexual activity aligned with peak fertility period was compared to those who partially or did not align to peak fertility period using Cox proportional hazards with Andersen–Gill extension and robust standard error estimates.

### Ethics

All participants provided written informed consent in their preferred language (English or Kiswahili). The study protocol was approved by ethical review boards at the University of Washington, Kenya Pharmacy and Poisons Board, and Kenya Medical Research Institute (KEMRI).

## Results

**Fig. 1:**
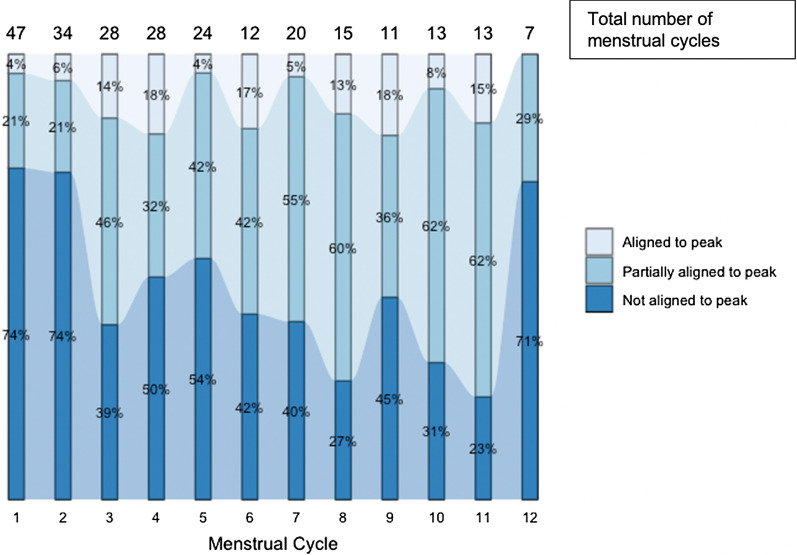
Proportion of women with alignment between condomless sex and peak fertility days, by menstrual cycle number

We included data from 6929 daily SMS surveys that were captured during 252 menstrual cycles of 65 women whose peak fertility windows were able to be calculated. There were 9 women excluded from analysis because they did not have predicted peak fertility periods (n = 3), became pregnant before their first predicted peak fertility period or SMS report (n = 5), or did not complete SMS reports (n = 1). Women had a median age of 30.1 years, had no children (interquartile range [IQR]: 0–1), and were in their partnerships for 2 years (IQR: 1–3 years). Approximately half of the women were living with HIV (47.7% and 53.3% were HIV-negative and in a partnership with a person living with HIV) and, of these women, two-thirds were virally suppressed (Table 1). Demographic characteristics of the excluded women were similar to the women included. Forty-six women (71%) achieved alignment during ≥ 1 menstrual cycle; condomless sex acts were reported during 24.3% (225/925) of peak fertility days and 11.3% (681/6004) of non-peak days (Chi-square p-value: < 0.0001). Women who aligned condomless sex to peak fertility at least once during the study reported more condomless sex acts both within peak fertility (median 3 vs. 0) and overall (median 11 vs. 1) than women who never aligned condomless sex and peak fertility days. When assessing alignment categories across menstrual cycle months, there was a large proportion of women who had condomless sex outside of the predicted peak fertility window in both not aligned and partially aligned categories (Fig. 1).

**Table 1:** Descriptive statistics overall and for women who ever achieved alignment or never achieved alignment across all menstrual cycles

	Ever alignment	Never alignment	Overall
	(N = 46)	(N = 18)	(N = 65)
Demographics			
Living with HIV, n (%)	23 (50.0%)	7 (38.9%)	31 (47.7%)
On ART/PrEP at baseline			
Virally suppressed (< 400 copies/mL)	15 (65.3%)	6 (54.5%)	21 (67.7%)
Initiated PrEP	23 (100%)	11 (100%)	34 (100%)
Age, years, median (IQR)	30.8 (27.9–35.5)	29.2 (26.3–36.0)	30.1 (27.8–35.8)
Partnership duration, years, median (IQR)	2 (2–3)	2 (1–3)	2 (1–3)
# prior children with study partner, median (IQR)	0 (0–1)	0 (0–1)	0 (0–1)
Time known to be discordant, years, median (IQR)	1 (0.5–3)	3 (0.25–5)	1 (0.5–4)
Drinks alcohol, per month, n (%)	4/242 (1.7%)	0/33 (0%)	4/280 (1.4%)
Social harm^a^	7/242 (2.9%)	2/33 (0.6%)	10/280 (3.6%)
STI diagnosis^b^	6/242 (2.5%)	0/33 (0%)	6/280 (2.1%)
Overall sexual activity			
# condomless sex acts per SMS reports, n (%)	877/6042 (14.5%)	29/865 (3.3%)	906/6929 (13.1%)
Median (IQR) condomless sex acts	11 (5–23)	1 (0–2)	7 (2–14)
# condomless sex acts during peak fertility per SMS report, n (%)	225/822 (27.4%)	0/103 (0%)	225/925 (24.3%)
Median (IQR) condomless sex acts during peak fertility	3 (1–7)	0 (0–0)	2 (0–4)

*ART* Antiretroviral therapy, *IQR* interquartile range, *PrEP* pre-exposure prophylaxis, *SMS* short message survey, *STI* sexually transmitted infections

^a^ Social harm refers to verbal, physical, or economic abuse by their study partner

^b^ STI diagnosis refers to trichomonas, gonorrhea, chlamydia tests completed at each visit

There were 5625 SMS surveys across 205 menstrual cycles of 52 women included for analysis of alignment of condomless sex and peak fertility. When assessing FAM indicators across peak fertility days, condomless sex was more frequent on days without menses, days with OPK+ status, and days with “sticky” cervical mucus (Table 2). Cervical mucus being “sticky” (adjusted odds ratio [aOR]: 2.25, 95% confidence interval [CI]: 1.30, 3.90) and positive OPK results (aOR: 2.07, 95% CI: 1.11, 3.86) were associated with greater likelihood of condomless sex during peak fertility days (Table 2). When assessing characteristics associated with being partially aligned versus not aligned across menstrual cycle months, age (odds ratio (OR):1.08, 95% CI: 1.01, 1.15) and not trying to become pregnant during this month (OR: 0.16, 95% CI: 0.08, 0.33) were associated with partial alignment to the peak fertility period. During the widened peak fertility period, not trying to become pregnant (OR: 0.15, 95% CI: 0.08, 0.31) was associated with partial alignment. When assessing characteristics associated with sexual activity being aligned versus not aligned to peak fertility days and using the wider definition of peak fertility days, number of prior children (OR: 0.62, 95% CI: 0.4, 0.98) was associated with achieving alignment (Table 3). Sensitivity analyses were performed to assess whether there was a meaningful effect of misclassification when matching menstrual cycle data to in-clinic interviews, and we found a similar magnitude and same direction for all associations.

**Table 2:** Associations between fertility indicators and condomless sex during peak fertility days (N = 52 women)

	Days with condomless sex/days with indicated fertility (%)	Days with condomless sex/ days without indicated fertility (%)	Unadjusted		Adjusted^a^	
			OR	p-value	OR	p-value
			95% CI		95% CI	
Peak fertility (N = 752)						
No menstruation	202/720 (28.1%)	4/30 (13.3%)	3.72	0.13	1.37	0.76
			0.67, 20.57		0.17, 10.78	
OPK+	45/99 (45.5%)	104/400 (26.0%)	2.85	**< 0.0001**	2.07	**0.02**
			1.78, 4.58		1.11, 3.86	
Cervical mucus+	79/181 (43.7%)	115/511 (22.5%)	3.08	**< 0.0001**	2.25	**0.004**
			2.0, 4.76		1.30, 3.90	
Peak fertility ± 2 days (N = 1470)						
No menstruation	323/1395 (23.2%)	4/72 (5.6%)	8.01	**0.0086**	3.01	0.13
			1.70, 37.80		0.72, 12.49	
OPK	68/156 (43.6%)	170/767 (22.2%)	2.86	**< 0.0001**	2.09	**0.0059**
			1.90, 4.20		1.24, 3.53	
Cervical mucus	118/321 (36.8%)	191/1018 (18.8%)	2.64	**< 0.0001**	2.09	**0.0003**
			1.84, 3.80		1.40, 3.13	

Bolded font indicates statistical significance at alpha = 0.05 level

^a^Adjusted for all other SMS predictors, respectively

**Table 3:** Unadjusted associations between participants’ characteristics and alignment of sexual behavior and peak fertility (N = 52 women)

	Median (IQR)			Aligned vs. Not aligned		Partial vs. Not aligned	
	N/Cycle-months (Total %)						
	Aligned	Partially aligned	Not aligned	OR (95% CI)	p-value	OR (95% CI)	p-value
**Demographic characteristics**							
Living with HIV	9/22	32/86	38/97	1.07	0.88	0.92	0.84
	40.9%	37.3%	39.2%	(0.42, 2.76)		(0.41, 2.08)	
Age, years	29.3	31.4	30.1	0.97	0.46	**1.08**	**0.02**
	(28.3–31.0)	(29.2–38.5)	(27.8–34.9)	(0.89, 1.05)		**(1.01, 1.15)**	
**Couple characteristics and fertility intentions**							
Partnership duration, years	2.0	2.0	2.0	0.91	0.73	1.2	0.48
	(2–2)	(2–3)	(2–3)	(0.55, 1.53)		(0.73, 1.97)	
Number of prior children with study partner	0	0	0	0.65	0.22	1.11	0.66
	(0–0)	(0–1)	(0–1)	(0.33, 1.29)		(0.70, 1.77)	
Time known to be discordant, years	1.0	1.0	1.0	0.97	0.79	1.05	0.45
	(0.4–4.0)	(0.75–4.0)	(0.4–4.0)	(0.77, 1.22)		(0.91, 1.22)	
Couple not trying to get pregnant this month	7/22	10/86	44/97	0.56	0.36	**0.16**	** < 0.0001**
	31.8%	11.6%	45.4%	0.16, 1.94		**0.08, 0.33**	
Ever used birth control	17/22	57/86	57/97	2.39	0.08	1.4	0.49
	77.3%	66.3%	58.8%	(0.91, 6.26)		(0.56, 3.41)	
***Peak fertility (± 2 days)***							
** Demographic characteristics**							
Female partner living with HIV	15/44	37/84	28/77	0.91	0.80	1.38	0.54
	34.1%	44.0%	36.4%	(0.42, 1.94)		(0.48, 3.92)	
Age, years	30.1	30.8	30.1	1.03	0.19	1.05	0.24
	(29.2–38.2)	(27.8–41.4)	(27.8–36.0)	(0.89, 1.10)		(0.97, 1.13)	
**Couple characteristics and fertility intentions**							
Partnership duration, years	2.0	2.0	3.0	0.73	0.73	1.24	0.45
	(1–2)	(2–3)	(2–3)	(0.42, 1.27)		(0.70, 2.18)	
Number of prior children with study partner	0	0	0	**0.62**	**0.04**	1.01	0.96
	(0–0)	(0–1)	(0–1)	**(0.40, 0.98)**		(0.63, 1.63)	
Time known to be discordant, years	1.0	1.5	1.0	0.87	0.19	1.06	0.47
	(0.5–1.0)	(0.75–4.0)	(0.33–4.0)	(0.71, 1.07)		(0.90, 1.25)	
Couple not trying to get pregnant this month	14/44	11/84	38/77	0.48	0.06	**0.15**	**< 0.0001**
	31.8%	13.1%	49.4%	(0.21, 1.05)		**(0.08, 0.31)**	
Ever used birth control	32/44	54/84	44/77	2.0	0.07	1.35	0.6
	72.7%	64.3%	57.1%	(0.93, 4.28)		(0.45, 4.08)	

Bolded font indicates statistical significance at alpha = 0.05 level

**Fig. 2:**
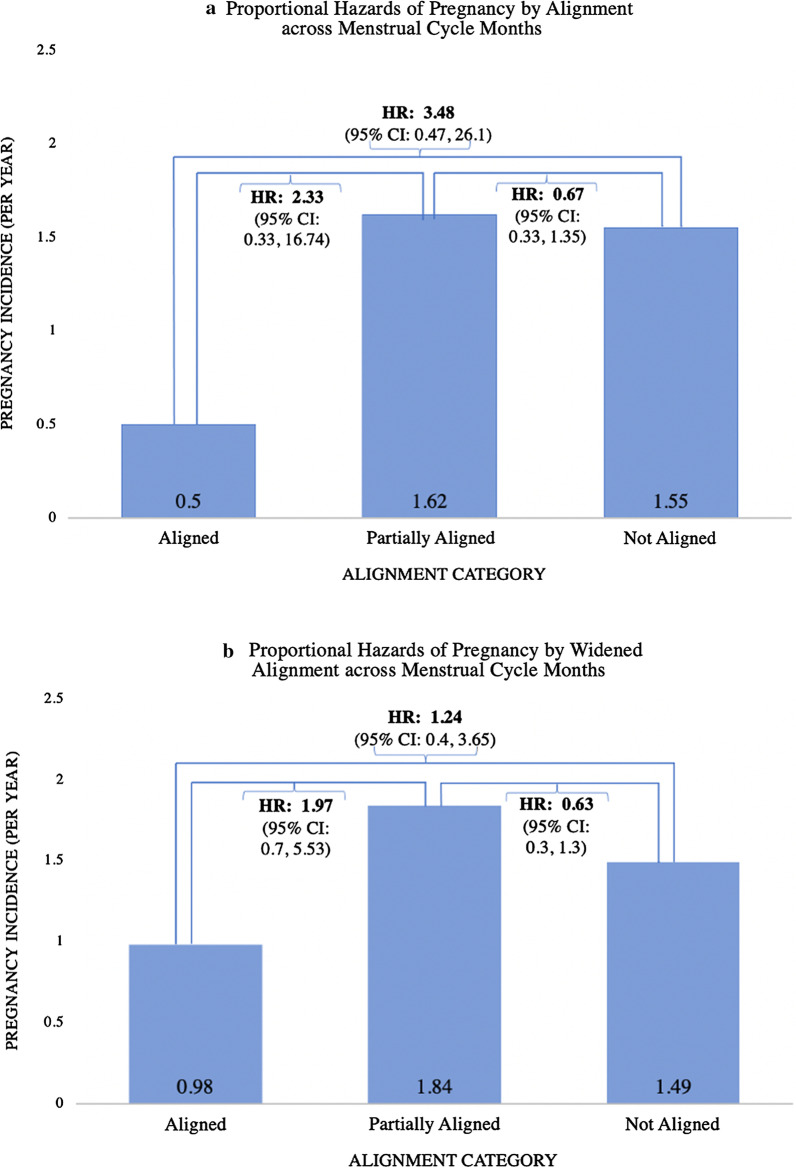
Proportional hazards of pregnancy by alignment across menstrual cycle months (n = 252 menstrual cycles). The hazard ratios (HR) compare pregnancy incidence among categories of sexual activity aligned with peak fertility period using Cox proportional hazards with Andersen–Gill extension

There were 31 pregnancies among women in our analysis. The pregnancy incidence rate was 0.5 pregnancies per year for aligned cycles, 1.62 for partially aligned cycles, and 1.55 for not aligned cycles. Using a widened definition of peak fertility (± 2 days), similar pregnancy incidence rate pattern was observed across alignment categories (Table 4). Across all SMS surveys, there were two women who always reported both OPK-negative and non-sticky cervical mucus statuses, and an additional four women reported OPK-negative status and four women reported non-sticky cervical mucus. In our multivariate model, it was not possible to adjust for HIV status since no women living with HIV became pregnant during an aligned cycle month. Sex aligned with peak fertility (as the referent category) was not associated with pregnancy incidence relative to periods with full non-alignment (hazard ratio [HR]: 2.33, 95% CI: 0.33, 16.74) and partial alignment (HR: 3.48, 95% CI: 0.47, 26.1) (Fig. 2a). When using a widened definition of peak fertility (± 2 days), sex aligned with peak fertility remained not associated with pregnancy incidence relative to periods with full non-alignment (HR: 1.24, 95% CI: 0.42, 3.65) and partial alignment (HR: 1.97, 95% CI: 0.7, 5.53) (Fig. 2b). Sex partially aligned with peak fertility was associated with a lower but insignificant difference in incidence of pregnancy relative to periods with non-alignment across both lengths of peak fertility (HR: 0.67, 95% CI: 0.33, 1.35 for the 3-day length and HR: 0.63, 95% CI: 0.3, 1.3 for the widened length).

**Table 4:** Pregnancy incidence by alignment category and menstrual cycles

	Pregnancies/menstrual cycles	Pregnancy incidence (per year)
**Peak fertility**		
Aligned	1/24	0.5
Not aligned	17/132	1.55
Partially aligned	13/96	1.62
**Widened peak fertility (± 2 days)**		
Aligned	4/49	0.98
Not aligned	13/105	1.49
Partially aligned	15/98	1.84

## Discussion

Among HIV-serodifferent couples engaged in a comprehensive safer conception program to minimize HIV transmission during pregnancy attempts, a moderate percentage (71%) of women aligned condomless sex to predicted peak fertility periods at least once. After training women and couples on the use of FAM indicators, there was high uptake of the use of FAM methods reported via SMS (65 of 74 couples). Across similar settings in Africa, couples and women desiring pregnancy are not often aware of safer conception methods or have low uptake of methods such as timed intercourse [20–23]. There were no HIV transmission events in this cohort [16].

Sticky cervical mucus and positive OPK results allow women to self-estimate their peak fertility period and reporting these characteristics was associated with timing of condomless sex in our cohort. Other studies have found that cervical mucus status is the strongest indictor of upcoming fertility days and that self-tracking mucus characteristics reduces the time-to-pregnancy [12, 24]. Our data are among the first from African women to demonstrate feasibility of tracking these indicators and alignment of fertility signs with condomless sex. This is key for resource limited settings where access to ultrasound, in vitro fertilization, and other measures to definitively assess ovulation are not accessible for many women.

Previous fecundity studies have demonstrated that tracking FAM improves fertility rates [1, 12, 13]. In SCIP, the parent study to this one, the 12-month cumulative pregnancy rate was 61.8% among all 74 couples and approximately half became pregnant [16]. This analysis shows that women with more menstrual cycle months where condomless sex was concurrent with peak fertility days did not have a significantly different pregnancy incidence rate than those who did not align or partially aligned sexual activity with predicted peak fertility. There were five couples who did not report condomless sex at any point and all couples reported using condoms at least once. There were also fewer menstrual cycles when condomless sex overlapped (24 cycles) with peak fertility than no overlap (132 cycles), indicating that condomless sex outside of peak fertility was a frequent occurrence, regardless of the woman’s HIV status. Similar patterns were observed across the widened peak fertility definition. Women who achieved pregnancy during an aligned month may have had similar patterns of concurrent sexual behaviors and fertility periods over time to women who did not achieve pregnancy during an aligned month, which could partially account for the lack of improvement in time to pregnancy. Qualitative analysis from SCIP participants indicated that couples often report challenges with condom use outside of the peak fertility period and that couples-based counseling provides a chance to support one another to practice safer conception [25]. In a prospective cohort of couples engaged in a safer conception program in South Africa, a lower proportion of women in HIV-serodifferent relationships used timed intercourse rather than self-insemination as compared to our analysis, perhaps indicating variance in preferences to use FAM indicators [26].

Our analysis had several limitations including that this was a small sample of couples attempting pregnancy and may not be broadly generalizable. Further, there may be misclassification of the time-varying characteristics since study visits were not timed to menstrual cycles and most data points for time-varying confounding factors straddled two consecutive cycles. We conducted sensitivity analyses to assess whether there was a meaningful effect of misclassification when matching menstrual cycle data to in-clinic interviews, and we found a similar magnitude and same direction for all associations. Finally, the pregnancy incidence rates may indicate misreporting of sexual activity or misclassification of fertility periods since the aligned definition of sex only within the peak fertility period had a lower pregnancy incidence than partial or non-aligned.

## Conclusions

Safer conception interventions improve reproductive health outcomes of heterosexual HIV-serodifferent couples attempting pregnancy and uphold the reproductive rights of HIV-serodifferent couples to satisfy their desires to attempt pregnancy [27]. The FAM indicators included in this study engaged couples with pregnancy planning and possibly reduced the number of condomless sex acts, thereby reducing HIV transmission risk. However, the processes involved in accurately identifying peak fertility can be complex, and require guidance and continuous support from healthcare providers to accompany this method and we didn’t see a quantifiable benefit in terms of pregnancy optimization. Thus, ART and PrEP are very important for HIV-serodifferent couples seeking to conceive, and may be more critical to promote than FAM indicators.

## Data Availability

The datasets used and/or analyzed during the current study are available from the corresponding author on reasonable request.

## Abbreviations

ART: Antiretroviral therapy
FAM: Fertility awareness methods
OPK: Ovulation prediction kit
PrEP: Pre-exposure prophylaxis
SMS: Short-message survey
